# Visual Loss after Scoliosis Surgery: What Do Surgeons and Patients Need to Know? Three Case Reports

**DOI:** 10.1055/s-0044-1791188

**Published:** 2024-12-27

**Authors:** Alderico Girão Campos de Barros, Augusto Ribeiro de Jesus Oliveira, Lucas Rocha Cavalvanti, Luis Eduardo Carelli Teixeira da Silva, João Antonio Matheus Guimarães

**Affiliations:** 1 Grupo de Cirurgia da Coluna, Instituto Nacional de Traumatologia e Ortopedia (INTO), Rio de Janeiro, RJ, Brasil; 2 Programa de Pós-graduação, Instituto Nacional de Traumatologia e Ortopedia (INTO), Rio de Janeiro, RJ, Brasil

**Keywords:** amaurosis, blindness, cortical, spine/surgery, scoliosis/complications

## Abstract

Visual deficit after spinal surgery is rare but tragic. The main causes include external eye injury, cortical blindness, central retinal artery occlusion, and ischemic optic neuropathy. In scoliosis surgery, this complication potentially arises from prolonged surgical time, high blood loss, prone position, hydroelectrolytic imbalance, and cerebrospinal fluid loss.

In 849 scoliosis correction surgeries, 3 patients developed postoperative visual deficits: 2 achieved complete visual acuity recovery, but 1 remained with partial sequelae.

There are four causes of postoperative amaurosis: ischemic optic neuropathy, central retinal artery occlusion, external eye injury, and cortical blindness. Since the prevention of this complication cannot be assured, it is essential to explain the risk of visual deficits to patients undergoing scoliosis surgery, who must sign the informed consent form.

Visual loss after spinal surgery for scoliosis correction is a rare but severe and, sometimes, irreversible complication. The surgical team must know about this possibility in order to adopt preventive measures and reduce its incidence.

## Introduction

Ophthalmologic complications after surgery are rare, with an incidence of approximately 1:125 thousand, and they are 75 times more frequent in patients undergoing cardiac surgery. 1 2 In spinal surgery patients, the first description of this complication occurred in 1950, and its estimated incidence ranges from 0.03 to 0.2%. 3 The main causes include external eye injury (EEI; corneal abrasion), cortical blindness (CB), central retinal artery occlusion, and ischemic optic neuropathy (ION). 4 Many risk factors predispose to this complication, such as prolonged surgery, high blood loss, prone position during the procedure, blood transfusion, arterial hypotension, hydroelectrolytic imbalance, and cerebrospinal fluid loss. 5 Patient-related factors, such as atherosclerosis, obesity, diabetes mellitus, collagen diseases, coagulopathies, systemic arterial hypertension, patent foramen ovale, peripheral vascular disease, smoking, and alcohol and drug abuse deserve consideration. 6 Other independent risk factors include male gender, obesity, use of a Wilson frame for patient positioning, longer surgical times, higher blood loss, and lower colloid-to-crystalloid ratio in blood fluid administration. Modifying some of these factors may reduce the risk of this complication, but its relatively low incidence, the ethical limitations regarding randomized studies, and the lack of a current animal model limit the evidence level. 7

## Report of three cases

In the 20-year survey period, from 2002 to 2022, 849 scoliosis correction surgeries were performed at our institution. An analysis of the medical records revealed this complication in the three following patients, who presented visual deficits in the immediate postoperative period:

### Case 1

We present the case of BDT, 13 years old, female, brown, weight of 36 Kg, body mass index (BMI) of 18.4 Kg/m ^2^ , non-smoker, non-alcoholic, and non-user of illicit drugs. She had left-sided thoracic scoliosis associated with the use of a chest tube at 3 months old due to pneumonia that had been diagnosed late. Preoperatively, her Cobb angle was of 120°, and the patient had a mild restrictive pulmonary disorder with no other known comorbidities and an American Society of Anesthesiologists (ASA) I classification. She underwent a T2-to-L3 thoracolumbar arthrodesis in 2 stages. The first surgical stage consisted of curve instrumentation with pedicle screws, periapical osteotomies, and cranial halo placement for intraoperative traction. The patient evolved well, and the 2nd surgical stage, 14 days after the 1st, consisted of curve correction with rods and an autologous graft. During surgery, general anesthesia was uneventful. The patient received one bag of packed red blood cells. The intraoperative surgical bleeding was of 600 mL, and wound drainage was of 400 mL. There was no hypotension, hypothermia, neither were there other signs of hemodynamic instability during the perioperative period. The total operative time was of 260 minutes. In the immediate postoperative period of the second surgical procedure, the patient presented bilateral amaurosis followed by difficult-to-control seizures and status epilepticus, requiring orotracheal intubation. Seizure control occurred after the administration of an attack phenytoin dose and phenobarbital. The team requested a contrast-enhanced computed tomography (CT) scan and a magnetic resonance imaging (MRI) scan of the skull, a Doppler of the carotid and vertebral arteries, and an electroencephalogram; all results were unremarkable. The patient underwent evaluation by a multidisciplinary team, and the ophthalmologic physical examination showed no retinal or pupillary alterations. After two days, the patient spontaneously recovered her vision, with no impairment to her visual acuity. The patient is under follow-up as an outpatient and remains stable.

### Case 2

We reported the case of CRC, 19 years old, female, brown, weight of 34.4 Kg, BMI of 15.7 Kg/m ^2^ , without comorbidities, and ASA I. She had congenital thoracic scoliosis, with a Cobb angle of 96° from T1 to T9 before surgery. The echocardiogram and ultrasound (US) of the genitourinary system were unremarkable, and the MRI scan of the spine showed no intraspinal alterations. Surgery consisted of a posterior vertebrectomy of T6, osteotomy, and curve correction, followed by posterior arthrodesis from T1 to L3 using an autologous spinous process graft. The surgery occurred under neurophysiological monitoring. The patient underwent general anesthesia. During the procedure, there was significant hemodynamic instability due to excessive bleeding and a global reduction in the neurophysiological signals measured. After strict control of the patient's hemostasis and volume expansion, the team reversed and stabilized the condition. The surgical bleeding was of 5 L, and the patient received 10 units of packed red blood cells, 4 platelet bags, 2 plasma bags, 2 albumin bags, 1 g of methylprednisolone, 8.5 L of crystalloid, and 1 L of colloid. Clinical complications, including consumption coagulopathy, bilateral pneumothorax, and pulmonary sepsis due to *Klebsiella* *sp* . occurred postoperatively. The patient remained on mechanical ventilation for 18 days, with anisocoria identification on the 1st day. A multidisciplinary team monitored the patient. Fundoscopy and CT of the skull were unremarkable. After the sedation period, a new ophthalmological evaluation revealed amaurosis on the left side. The patient was under follow-up for 2 years, showing partial visual field improvement, but remained with visual loss-related restrictions.

### Case 3

**Fig. 1:**
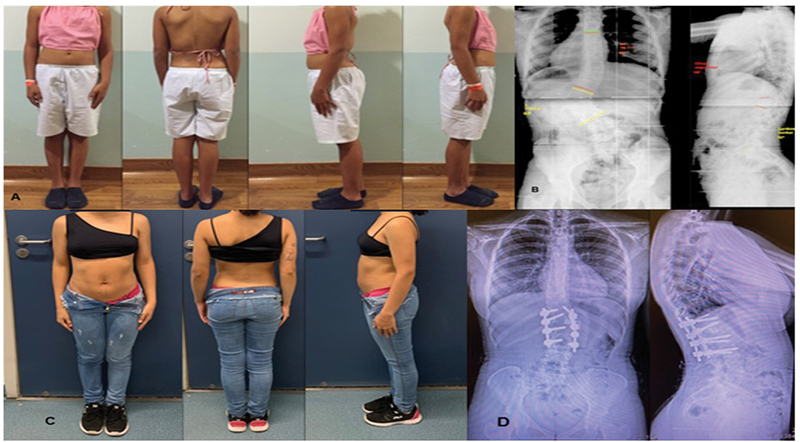
Case 3: Congenital scoliosis treated with L1 hemivertebrectomy and T11–L3 posterior arthrodesis. ( **A** ) Preoperative clinical images. ( **B** ) Preoperative radiographs. ( **C** ) Five-year follow-up clinical images. ( **D** ) Five-year follow-up radiographs.

We present the case of LSSS, 14 years old, female, weight of 36 Kg, without comorbidities, and ASA I. She had congenital scoliosis, with hemivertebra in L1 and a Cobb angle of 68° (T12–L3) in the preoperative period. A preoperative transthoracic echocardiogram showed mild mitral insufficiency, a urinary tract US revealed dilation of the right renal pelvis, and an MRI scan of the spine showed no intraspinal changes. During surgery, the surgeon performed L1 hemivertebra via a posterior approach, osteotomy, and curve correction, followed by posterior arthrodesis from T11 to L3 with an autologous graft. The surgery occurred under neurophysiological monitoring, and the final responses were similar to the initial ones. The procedure required the transfusion of 1 unit of packed red blood cells (packed cell volume: 23%). The intraoperative bleeding was of approximately 500 mL, and the patient did not present hemodynamic instability or neurophysiological sign alteration. The total operative time was of 270 minutes. Seven hours after the end of the procedure, in the pediatric intensive care unit, the patient presented a generalized tonic-clonic seizure lasting less than 5 minutes and received anticonvulsant medication. Although she recovered consciousness in approximately 10 minutes, she developed bilateral amaurosis, with complete deficit recovery 12 hours after its onset. The team requested a CT scan, an MRI scan of the skull, and an electroencephalogram. All tests, including the ophthalmological examination, were unremarkable ( Fig. 1 ).

## Discussion

The following four conditions are the main causes of decreased visual acuity after spinal surgery:

### Ischemic Optic Neuropathy

**Fig. 2:**
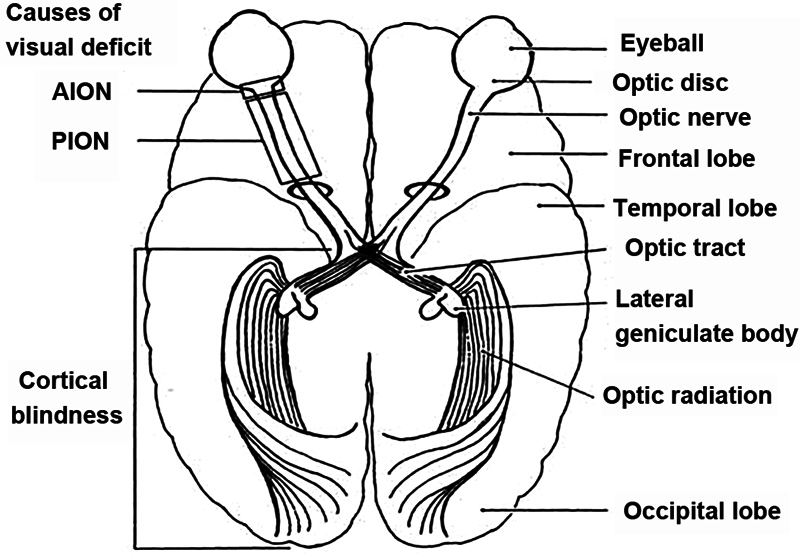
Main sites of abnormalities leading to postoperative visual loss and its different causes. **Abbreviations:** AION, anterior ischemic optic neuropathy; PION, posterior ischemic optic neuropathy (adapted from Williams et al., 1995 ^11^ ).

Resulting from an imbalance between oxygen supply and demand in the optic nerve, which damages the nerve fibers, ION can be anterior (AION) or posterior (PION). The impairment is often very severe and bilateral due to irreparable optic nerve damage, regardless of whether the injury occurred in the optic disc (AION) or the retrobulbar optic nerve (PION). 8 The condition may be unilateral or bilateral, appearing immediately after surgery or in a few days. To date, there is no proven beneficial treatment, and visual recovery is usually poor. The posterior region is the area most commonly affected due to the its peculiar vascularization. The posterior optic nerve receives blood from vessels arising from the optic artery, which have poor autoregulation, making the nerve vulnerable to anemia and hypotension. 9 Posterior ION presents as painless visual loss upon awakening from anesthesia. It often does not progress, but recovery is poor, and no treatment has been proven effective. In turn, prolonged prone positioning can cause postoperative facial or periorbital edema, leading to indirect elevation of orbital venous pressures and contributing to ischemia. Headrest use and constant head position monitoring during surgery have been shown to reduce this complication 8 ( Fig. 2 ).

### Central Retinal Artery Occlusion

Central retinal artery occlusion results from decreased blood supply to the entire retina. 7 It is associated with hypercoagulability states (causing embolism-related injury) or external eyeball compression. This compression may occur, for instance, due to the prone position during spinal surgeries, which increases intraocular pressure and occludes the internal retinacular circulation. Retinal ischemia clinically manifests as decreased pupillary reflex and a “cherry red” spot in the macula visible on fundoscopic examination. Postoperative improvement is low, and there is no adequate treatment for this complication. 8

### External Eye Injury

External eye injury presents as corneal ulceration or irritation resulting from direct trauma related to the prone position, predisposing to infection and local inflammation. The factors related to EEI include the prone position, Trendelenburg position, prolonged operative time, and obesity. Positioning is the most controllable risk factor. Recommendations to minimize the EEI risk include positioning the patient in ventral decubitus, adopting 10° to 15° of reverse Trendelenburg, ocular occlusion with a proper lubricant, and facial support that releases the periocular region. 1 10

### Cortical Blindness

A rare clinical condition, CB is characterized by low visual acuity resulting from injury to the retrogeniculate pathways or visual cortex. 1 It usually manifests upon awakening from anesthesia. 6 Stroke is its main cause, followed by embolic and other systemic conditions. 2 3 The main risk factors are systemic arterial hypertension, diabetes mellitus, hypercholesterolemia, heart disease, atheromatous diseases of the neck vessels, smoking, use of oral contraceptives, hormone replacement therapy, and stress. 1 The diagnosis relies on decreased visual acuity and visual field abnormalities associated with infarction areas, mainly in the region of the posterior cerebral artery, confirmed by CT or MRI scans. 7 8

**Fig. 3:**
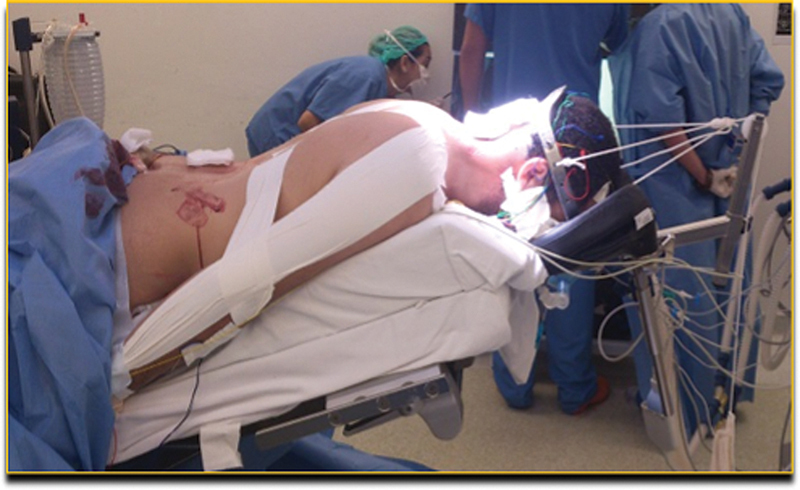
Prone positioning with attention to the correct head position and use of support. One must pay attention to changes in position during the procedure in conjunction with motor stimulation of neuromonitoring.

Visual changes after spinal surgery negatively impact the patient's quality of life; an observational study 7 reported that 86% of patients undergoing spinal surgery in the prone position preferred to receive information about the risk of visual loss. Recognizing and adequately managing the risk factors and diagnosing complications early are essential to prescribe potentially-effective therapies. Although hypotensive anesthesia prevents excessive blood loss during spinal surgery, the cases herein reported have suggested that it may be a significant risk factor for visual impairment; further research on the optimal blood pressure range during surgery is warranted. Patients and physicians must assess and recognize the benefits and risks of this strategy. 10 The proper prone positioning of the patient requires special attention 1 2 ( Fig. 3 ). In the case series herein presented, bilateral amaurosis in the first and third patients was part of the postoperative convulsive symptoms. The prognosis of CB depends on the cause, severity, and duration of the triggering factor, 5 which explains why amaurosis is transient and patients recover quickly. Other combined risk factors, such as hemodynamic instability, prolonged operative time, high blood loss, and multiple transfusions of blood products and crystalloids indicate ischemia of multifactorial etiology in the second patient. There is evidence that up to 94% of the cases of optic nerve neuropathy occurred when anesthesia time exceeded 6 hours and blood loss was higher than 1 L. A review study 11 indicated a strong association involving intraoperative arterial hypotension and anemia with postspinal surgery amaurosis. The ASA, along with spinal surgeons and neuro-ophthalmologists, developed a practical manual to prevent perioperative ophthalmological complications in spinal surgery. 10 11 12 13 14 Chart 1 lists the main recommendations. However, it is worth noting that these recommendations do not apply to CB. 10 Since there is no way to assure its prevention, it is critical to explain the risk of visual deficit to patients who must undergo prolonged spinal surgeries in the prone position, procedures with the expectation of substantial blood loss, or both; these patients must sign the informed consent form.

**Chart 1:** Practice recommendations of the American Society of Anesthesiologists (2019) to manage patients at high risk for spinal surgery-related visual loss

• Consider continuous blood pressure and central venous pressure monitoring.
• Avoid direct pressure on the eyeball.
• Positioning for high-risk patients includes placing the head at the same level (or above) the rest of the body when possible. If feasible, the patient's head must be in a neutral forward position (such as with no significant neck flexion, extension, lateral flexion, or rotation).
• Maintain blood pressure at higher levels in hypertensive patients to prevent end-organ risk. Only use deliberate hypotension in high-risk patients when the anesthesiologist and surgeon agree that it is essential.
• Periodically monitor the levels of hemoglobin or packed cell volume during surgery. The transfusion threshold that may decrease the risk of injury is unknown.
• Use colloids and crystalloids to maintain intravascular volume in patients with excessive blood loss.
• Additional management may include optimization of the levels of hemoglobin or packed cell volume, hemodynamic status, and arterial oxygenation.
• Consider procedures in stages for high-risk patients.
• Perform an ophthalmologic examination as soon as the patient becomes alert. If visual loss is a possibility, request an urgent ophthalmologic consultation.

**Note:** High-risk patients include those undergoing prolonged procedures with substantial blood loss.

Visual loss after spinal surgery for scoliosis correction is a rare but severe and sometimes irreversible complication. The surgical team must know about it to adopt preventive measures and reduce its incidence.
