# Supplementary material for: Visual Loss after Scoliosis Surgery: What Do Surgeons and Patients Need to Know? Three Case Reports
Source: Rev Bras Ortop (Sao Paulo). 2024 Dec 27;59(Suppl 2):e259–63. doi: 10.1055/s-0044-1791188 (PMC11679631; doi:10.1055/s-0044-1791188)
Supplement: Related texts

# Research article: Perda visual após cirurgia de escoliose: O que o cirurgião e os pacientes precisam saber? Relato de três casos

**Keywords:** amaurose, cegueira cortical, coluna vertebral/cirurgia, escoliose/complicações

## Abstract

Déficit visual após à cirurgia da coluna vertebral é raro, porém trágico. As principais causas incluem lesão ocular externa, cegueira cortical, oclusão da artéria central da retina e neuropatia óptica isquêmica. Na cirurgia de escoliose, o tempo prolongado, as grandes perdas sanguíneas, a posição em decúbito ventral, o desequilíbrio hidroeletrolítico e a perda de liquor estão associados a essa complicação.

Em 849 cirurgias de correção de escoliose, 3 pacientes evoluíram com déficit visual no pós-operatório, e 2 obtiveram recuperação completa da acuidade visual, mas 1 permaneceu com sequela parcial.

Existem quatro causas de amaurose pós-cirúrgica: neuropatia óptica isquêmica, oclusão da artéria central da retina, lesão ocular externa e cegueira de origem cortical. Como a prevenção dessa complicação não pode ser garantida, é fundamental que se esclareça o risco de déficit visual para os pacientes que serão submetidos à cirurgia de escoliose, que devem assinar o termo de consentimento livre e esclarecido.

A perda visual após cirurgia da coluna vertebral para correção de escoliose é uma complicação rara, porém grave e, por vezes, irreversível. É fundamental que a equipe cirúrgica saiba de sua existência para adotar medidas preventivas e, consequentemente, reduzir a sua incidência.

## Introdução

Complicações oftalmológicas após cirurgia são muito raras, com uma incidência aproximada de 1:125 mil, e são 75 vezes mais frequentes em pacientes submetidos a cirurgias cardiológicas. 1 2 Nos pacientes submetidos à cirurgia da coluna vertebral, essa complicação foi primeiramente descrita em 1950, e estima-se que sua incidência varie de 0,03 a 0,2%. 3 As principais causas incluem lesão ocular externa (LOE; abrasão de córnea), cegueira de origem cortical (COC), oclusão da artéria central da retina e neuropatia óptica isquêmica (NOI). 4 Muitos fatores de risco predispõem à ocorrência dessa complicação: cirurgias prolongadas, grandes perdas sanguíneas, posição em decúbito ventral durante o procedimento, transfusão sanguínea, hipotensão arterial, desequilíbrio hidroeletrolítico e perda de liquor. 5 Fatores relacionados ao paciente, como presença de aterosclerose, obesidade, diabetes mellitus, doenças do colágeno, coagulopatias, hipertensão arterial sistêmica, patência de forame oval, doença vascular periférica, tabagismo, e abuso de álcool e drogas também devem ser considerados. 6 Outros fatores de risco independentes e significativos incluem sexo masculino, obesidade, uso de armação de Wilson no posicionamento do paciente, tempos operatórios mais longos, maior perda de sangue e menor proporção de coloide para cristaloide na administração de fluido sanguíneo. A modificação de alguns desses fatores pode reduzir o risco dessa complicação, mas a incidência relativamente baixa, as limitações éticas para a realização de estudos randomizados e a falta de um modelo animal atual limitam o nível de evidência nos estudos. 7

## Relato de Três Casos

Num período de 20 anos de levantamento, entre 2002 e 2022, 849 cirurgias de correção de escoliose foram realizadas em nossa instituição. A análise dos prontuários médicos revelou a descrição da complicação em três pacientes, que apresentaram déficit visual no pós-operatório imediato, a saber:

## Caso 1

Apresentamos o caso de BDT, de 13 anos, do sexo feminino, parda, de 36 Kg, com índice de massa corpórea (IMC) de 18,4 Kg/m ^2^ , não tabagista, não etilista e não usuária de drogas ilícitas. Ela era portadora de escoliose toracogênica à esquerda associada ao uso de dreno torácico aos 3 meses de idade devido a uma pneumonia diagnosticada tardiamente. No pré-operatório, o ângulo de Cobb medido foi de 120°, a paciente apresentava distúrbio restritivo pulmonar leve, sem outras comorbidades conhecidas, e seu grau na classificação da American Society of Anesthesiologists (ASA) foi I. Ela foi submetida à artrodese toracolombar de T2 a L3 em 2 tempos. No primeiro tempo cirúrgico, foi realizada instrumentação da curva com parafusos pediculares, osteotomias periapicais e instalação de halo craniano para o uso de tração intraoperatória. A paciente evoluiu bem e, após 14 dias, foi realizado o segundo tempo cirúrgico, quando a curva foi corrigida por meio da colocação das hastes e enxertia autóloga. No ato cirúrgico, não houve intercorrências na anestesia geral, foi infundida uma bolsa de concentrado de hemácias, o sangramento cirúrgico intraoperatório foi de 600 mL, e a drenagem da ferida foi de 400 mL. Não houve hipotensão, hipotermia ou outro sinal de instabilidade hemodinâmica no perioperatório. O tempo cirúrgico total foi de 260 minutos. No pós-operatório imediato do segundo tempo cirúrgico, a paciente apresentou amaurose bilateral seguida de episódios convulsivos de difícil controle e estado de mal epilético, com necessidade de intubação orotraqueal. Após a hidantalização e o uso de fenobarbital, a convulsão foi controlada. Foram realizadas tomografia computadorizada (TC) com contraste e ressonância magnética (RM) de crânio, Doppler de artérias carotídeas e vertebrais e eletroencefalograma, todos normais. A paciente passou por avaliação de equipe multidisciplinar, e o exame físico oftalmológico não apresentava alteração retiniana ou pupilar. Após dois dias, a paciente recuperou espontaneamente a visão, sem qualquer prejuízo na acuidade visual, e está sendo acompanhada ambulatorialmente, com quadro estabilizado.

## Caso 2

Relatamos o caso de CRC, de 19 anos, do sexo feminino, de 34,4 Kg, IMC de 15,7 Kg/m ^2^ , parda, sem comorbidades, ASA I. Ela era portadora de escoliose torácica congênita, ângulo de Cobb de 96°, de T1 a T9 no pré-operatório. O ecocardiograma e a ultrassonografia (USG) do aparelho geniturinário não mostravam alterações, e a RM da coluna vertebral não apresentava alterações intraraquianas. No ato operatório, foi realizada a vertebrectomia de T6 por via posterior, osteotomia e correção da curva, seguida de artrodese posterior de T1 a L3 com uso de enxerto autólogo de processo espinhoso. A cirurgia foi realizada sob monitoramento neurofisiológico. A paciente foi submetida a anestesia geral. Durante o procedimento, houve importante instabilidade hemodinâmica por sangramento excessivo. No transcorrer da cirurgia, foi verificada redução global dos sinais neurofisiológicos aferidos e, após o rigoroso controle da hemostasia e a expansão volêmica da paciente, o quadro foi revertido e estabilizado. O sangramento cirúrgico foi de 5 L, e foi realizada transfusão de 10 concentrados de hemácias, 4 bolsas de plaqueta, 2 bolsas de plasma, e 2 bolsas de albumina; foram infundidos 1 g de metilprednisolona, 8,5 L de cristaloide e 1 L de coloide. No pós-operatório, ocorreram complicações clínicas como coagulopatia de consumo, pneumotórax bilateral e sepse pulmonar por *Klebsiella sp* . A paciente permaneceu sob ventilação mecânica por 18 dias e, no primeiro dia, foi identificada anisocoria. A paciente foi acompanhada por equipe multidisciplinar. A fundoscopia e a TC de crânio estavam normais. Após o período de sedação, nova avaliação oftalmológica foi realizada, e identificou-se amaurose à esquerda. A paciente foi acompanhada por 2 anos e apresentou melhora parcial do campo visual, mas permaneceu com restrições relacionadas à perda visual.

## Caso 3

Apresentamos o caso de LSSS, de 14 anos, do sexo feminino, de 36 Kg, sem comorbidades, ASA I. Ela era portadora de escoliose congênita, com hemivértebra em L1, e ângulo de Cobb de 68° (T12–L3) no pré-operatório. O ecocardiograma transtorácico pré-operatório apresentava insuficiência mitral leve, a USG de vias urinárias apresentava dilatação da pelve renal direita, e a RM da coluna vertebral não apresentava alterações intrarraquidianas. No ato operatório, foi realizada a ressecção da hemivértebra de L1, por via posterior, osteotomia e correção da curva, seguida de artrodese posterior de T11 a L3 com enxerto autólogo. A cirurgia foi realizada sob monitoramento neurofisiológico, com respostas finais semelhantes às iniciais. No procedimento, foi necessária a transfusão de 1 concentrado de hemácias (hematócrito [HT]: 23%), o sangramento no intraoperatório foi de aproximadamente 500 mL, e a paciente não apresentou instabilidade hemodinâmica ou alteração dos sinais neurofisiológicos. O tempo cirúrgico total foi de 270 minutos. Sete horas após o fim do procedimento, a paciente apresentou, em unidade de terapia intensiva pediátrica, crise convulsiva tônico-clônica generalizada com duração inferior a 5 minutos, e foi administrado anticonvulsivante. A paciente recuperou o nível de consciência em aproximadamente 10 minutos, mas evoluiu com amaurose bilateral, com recuperação completa do déficit 12 horas após o início dos sintomas. Foram realizados TC de crânio, RM de crânio e eletroencefalograma, todos sem alterações, e o exame oftalmológico encontrava-se já normal ( Fig. 1 ).

**Fig. 1:** Caso 3: escoliose congênita tratada com hemivertebrectomia de L1 e artrodese posterior de T11 a L3. ( **A** ) Imagens clínicas pré-operatórias. ( **B** ) Radiografias pré-operatórias. ( **C** ) Imagens clínicas de seguimento de 5 anos. ( **D** ) Radiografias de seguimento de 5 anos.

## Discussão

Quatro patologias são apontadas como as principais causas de diminuição da acuidade visual após cirurgia da coluna vertebral:

## Neuropatia Óptica Isquêmica

Resultado do desequilíbrio entre a oferta e a demanda de oxigênio no nervo óptico, NOI causa lesão às fibras nervosas, e pode ser dividida em NOI anterior (NOIA) ou posterior (NOIP). O comprometimento é muitas vezes muito grave e bilateral pelo dano irreparável do nervo óptico, independentemente de a lesão ocorrer no disco óptico (NOIA) ou no nervo óptico retrobulbar (NOIP). 8 A apresentação pode ser unilateral ou bilateral imediatamente após a cirurgia ou após alguns dias. Até o momento, não há tratamento comprovadamente benéfico, e a recuperação da visão geralmente é ruim. A região posterior é a área mais comumente afetada, devido à sua vascularização peculiar. O suprimento sanguíneo do nervo óptico posterior depende de vasos originados na artéria óptica, que têm autorregulação deficiente, o que deixa o nervo vulnerável à anemia e à hipotensão. 9 A NOIP geralmente se apresenta como perda indolor da visão ao acordar da anestesia, e normalmente não progride, mas a recuperação é ruim, e nenhum tratamento demonstrou eficácia. Por sua vez, o posicionamento prono prolongado pode causar edema facial ou periorbitário no pós-operatório, o que resulta na elevação indireta das pressões venosas orbitais e contribui para a isquemia. O uso de apoios de cabeça e a verificação constante da posição da cabeça durante a cirurgia comprovadamente reduziu essa complicação 8 ( Fig. 2 ).

**Fig. 2:** Principais locais de alterações responsáveis por perda visual pós-operatória em suas diferentes causas. **Abreviaturas:** NOIA, neuropatia óptica isquêmica anterior; NOIP, neuropatia óptica isquêmica posterior (adaptado de Williams et al., 1995 ^11^ ).

## Oclusão da Artéria Central da Retina

A oclusão da artéria central da retina é causada pela diminuição do suprimento de sangue para toda a retina. 7 Está associada a estados de hipercoagulabilidade, o que gera lesão por embolia ou ainda por compressão externa do globo ocular, devido, por exemplo, ao posicionamento em decúbito ventral durante as cirurgias de coluna, o que aumentará a pressão intraocular, e, por sua vez, ocluirá a circulação retinacular interna. A isquemia da retina se manifesta clinicamente com diminuição do reflexo pupilar e mancha “vermelho cereja” na mácula visível ao exame do fundo de olho. O grau de melhora no pós-operatório é baixo, e ainda não há ainda tratamento adequado para essa complicação. 8

## Lesão Ocular Externa

A LOE se apresenta como ulceração ou irritação da córnea decorrente de traumatismo direto relacionado ao decúbito ventral, o que predispõe a infecções e a inflamação local. Os fatores relacionados à LOE são o decúbito ventral, a posição de Trendelenburg, o tempo cirúrgico prolongado e a obesidade. O posicionamento é o fator de risco mais controlável. Recomendações para minimizar o risco desse tipo de lesão foram elaboradas: posicionar o paciente em decúbito ventral, adotar de 10° a 15° de Trendelenburg reverso, oclusão ocular com o uso de lubrificante apropriado e apoio facial que libere a região periocular. 1 10

## Cegueira de Origem Cortical

A COC é uma condição clínica rara, caracterizada por baixa acuidade visual, causada por lesão das vias retrogeniculadas ou do córtex visual. 1 Geralmente se manifesta ao despertar da anestesia. 6 Acidente vascular cerebral é a principal causa dessa condição, seguido de causas embólicas e outras patologias sistêmicas. 2 3 Os principais fatores de risco são hipertensão arterial sistêmica, diabetes mellitus, hipercolesterolemia, cardiopatias, doenças ateromatosas dos vasos do pescoço, tabagismo, uso de anticoncepcionais orais, reposição hormonal e estresse. 1 O diagnóstico é estabelecido pela diminuição da acuidade visual e pelas alterações do campo visual, associadas à presença de áreas de infarto, principalmente no território da artéria cerebral posterior, confirmada por TM ou RM. 7 8

Alterações visuais após cirurgias da coluna vertebral impactam negativamente a qualidade de vida do paciente, e um estudo observacional 7 relatou que 86% dos pacientes submetidos à cirurgia da coluna em decúbito ventral preferem ser informados do risco de perda visual. Reconhecer e controlar adequadamente seus fatores de risco, bem como diagnosticar precocemente a complicação, são fundamentais para a instituição de terapêuticas que possam ser eficazes. Embora a anestesia hipotensora seja usada para prevenir a perda excessiva de sangue durante a cirurgia da coluna, os casos aqui relatados sugeriram que pode ser um importante fator de risco para lesão visual, e pesquisas futuras sobre a faixa ideal de pressão arterial durante a cirurgia são justificadas. Os benefícios e riscos de tal estratégia devem ser avaliados e reconhecidos pelos pacientes e médicos. 10 Atenção especial deve ser dada ao posicionamento adequado do paciente quando em posição prona 1 2 ( Fig. 3 ). Na nossa casuística, a amaurose bilateral no primeiro e no terceiro casos fizeram parte do quadro convulsivo apresentado no pós-operatório. O prognóstico da COC depende da causa, da gravidade e da duração do fator desencadeante, 5 o que explica a transitoriedade da amaurose, com rápida recuperação dos pacientes. Outros fatores de risco combinados, como instabilidade hemodinâmica, tempo cirúrgico prolongado, grande perda sanguínea e politransfusão de hemoderivados e cristaloides evidenciam uma isquemia de etiologia multifatorial no segundo caso. Há evidência de que até 94% dos casos de neuropatia do nervo óptico ocorreram quando o tempo anestésico ultrapassou 6 horas e a perda sanguínea foi superior a 1 L. Um estudo de revisão 11 apontou forte associação entre hipotensão arterial transoperatória e anemia com a ocorrência de amaurose após cirurgia de coluna. A ASA desenvolveu, junto com cirurgiões de coluna e neuro-oftalmologistas, um manual prático para a prevenção das complicações oftalmológicas perioperatórias em cirurgia da coluna, 10 11 12 13 14 e as principais recomendações estão listadas no Quadro 1 . No entanto, é importante notar que essas recomendações não se aplicam à COC. 10 Como a prevenção dessa complicação não pode ser garantida, é fundamental que se esclareça o risco de déficit visual para os pacientes que devem ser submetidos a operações prolongadas da coluna em decúbito ventral e/ou procedimentos com expectativa de perda substancial de sangue; esses pacientes devem assinar o termo de consentimento livre e esclarecido.

**Quadro 1:** Recomendações práticas da American Society of Anesthesiologists (2019) para manejo de pacientes de alto risco para perda visual relacionada à cirurgia da coluna

• Deve-se considerar o monitoramento contínuo da pressão arterial e da pressão venosa central.
• Evitar pressão direta sobre o globo ocular.
• O posicionamento para pacientes de alto risco inclui colocar a cabeça no mesmo nível (ou acima) do resto do corpo quando possível. A cabeça do paciente também deve ser mantida em posição neutra para a frente (por exemplo, sem flexão significativa do pescoço, extensão, flexão lateral ou rotação) quando possível.
• Manter a pressão arterial em níveis mais elevados em pacientes hipertensos para prevenir riscos aos órgãos-alvo. Usar hipotensão deliberada apenas em pacientes de alto risco quando o anestesiologista e o cirurgião concordam que seu uso é essencial.
• Os valores de hemoglobina ou hematócrito devem ser monitorados periodicamente durante a cirurgia. O limiar de transfusão que diminuiria o risco de lesão ainda é desconhecido.
• Tanto os coloides quanto os cristaloides devem ser usados para manter o volume intravascular em pacientes com perda excessiva de sangue.
• O manejo adicional pode incluir a otimização dos valores de hemoglobina ou hematócrito, estado hemodinâmico e oxigenação arterial.
• Procedimentos em estágios devem ser considerados para pacientes de alto risco.
• Realizar exame oftalmológico assim que o paciente ficar alerta. Se houver possibilidade de perda visual, providenciar consulta oftalmológica urgente.

Nota: Pacientes de alto risco incluem pacientes que se submetem a procedimentos prolongados ou têm perda de sangue substancial.

**Fig. 3:** Posicionamento em decúbito ventral com atenção para a posição correta da cabeça e uso de apoio. Deve-se atentar para a mudança de posição durante o procedimento, em observância conjunta com o estímulo motor do neuromonitoramento.

A perda visual após cirurgia da coluna vertebral para a correção de escoliose é uma complicação rara, porém grave e, por vezes, irreversível. É fundamental que a equipe cirúrgica saiba de sua existência para adotar de medidas preventivas e, consequentemente, reduzir a sua incidência.
